# Supplementary material for: Peptide Stapling Improves the Sustainability of a Peptide-Based Chimeric Molecule That Induces Targeted Protein Degradation
Source: Int J Mol Sci. 2021 Aug 16;22(16):8772. doi: 10.3390/ijms22168772 (PMC8396023; doi:10.3390/ijms22168772)
Supplement: Supplementary file 1 [file ijms-22-08772-s001.zip › ijms-1322041-supplementary.pdf]

Supporting Information

Peptide Stapling Improves the Sustainability of a Peptide-Based Chimeric Molecule that Induces Targeted Protein Degradation

Hidetomo Yokoo[1,3,†], Nobumichi Ohoka[2,†], Mami Takyo[1,4], Takahito Ito[1], Keisuke Tsuchiya[1,5], Takashi Kurohara[1], Kiyoshi Fukuhara[5], Takao Inoue[2], Mikihiko Naito[6] and Yosuke Demizu[1,4,]*

[1] *Division of Organic Chemistry, National Institute of Health Sciences, 3–25–26, Tonomachi, Kawasaki, Kanagawa, 210–9501, Japan*

[2] *Division of Molecular Target and Gene Therapy Products, National Institute of Health Sciences, 3–25–26, Tonomachi, Kawasaki, Kanagawa, 210–9501, Japan*

[3] *Medical Chemistry, Graduate School of Medical Science, Kyoto Prefectural University of Medicine, Kyoto 606–0823, Japan*

[4] *Graduate School of Medical Life Science, Yokohama City University, Yokohama, Kanagawa, 230–0045, Japan*

[5] *Graduate School of Pharmacy, Showa University, Tokyo 142–0064, Japan*

[6] *Laboratory of Targeted Protein Degradation, Graduate School of Pharmaceutical Sciences, The University of Tokyo, Tokyo 113–0033, Japan*

[*] *Correspondence: demizu@nihs.go.jp; Tel.: +81–44-270–6578*

[†] *These authors contributed equally to this work.*

Table of Contents

Title	S1
Characterization of Peptides	S2
Figures S1 and S2	S10

Experimental Procedure

Synthesis of peptides

Method A : Fmoc-protected amino acids (5 eq), diisopropylcarbodiimide (10 eq), Oxyma pure (10 eq) in DMF were used each coupling. The residues of Arg and (*S*)-pentenyl alanine were performed additional coupling using Fmoc-protected amino acids (6 eq), diisopropylcarbodiimide (6 eq), Oxyma pure (6 eq) in DMF. Each coupling was performed under microwave irradiation and temperature was 15 seconds at 75˚C, then 110 seconds at 90˚C. Deprotection of Fmoc group was performed using 20% piperidine in DMF under microwave irradiation at 75˚C for 15 seconds, then at 90˚C for 50 seconds.
Method B : Fmoc-protected amino acids (6 eq), HBTU (6 eq), HOBt (6 eq), DIPEA(10 eq) in DMF were used each coupling. Each coupling was performed twice at 30℃ for 1 hr. Deprotection of Fmoc group was performed using 40% piperidine in DMF at 30℃ for 3 min, then 20% piperidine in DMF at 30℃ for 12 min.

Synthesis of PERML

After the peptide synthesis of **PERML** moiety (50 μmol scale) following Method A, the resin was suspended in cleavage cocktail [2 mL TFA, 50 μL water, 50 μL 1,2-ethanedithiol, 20 μL triisopropylsilane; final concentration: 94% TFA, 2.5% water, 2.5% 1,2-ethanedithiol, 1% triisopropylsilane] for 2 hr at rt. The TFA solution was evaporated to a small volume under a stream of N_2 and dripped into cold ether to precipitate the peptides.

Synthesis of PERML-R7

Employing the procedure described above for **PERML** and starting from **PERML-R7**.

Synthesis of stPERML

After the synthesis of peptide (50 μmol scale) following Method B without deprotecting N-terminus Fmoc group, ring-closing metathesis reactions were performed three times using 20 mol% second-generation Grubbs catalyst in DCE under microwave irradiation and temperature was at 70˚C for 12 min. Final deprotection was performed 4 mL of 20% PPD in DMF at rt for 10 min.

The resin was suspended in cleavage cocktail [2 mL TFA, 50 μL water, 50 μL triisopropylsilane; final concentration: 94% TFA, 2.5% water, 2.5% 1,2-ethanedithiol, 1% triisopropylsilane] for 2 hr at rt. The TFA solution was evaporated to a small volume under a stream of N_2 and dripped into cold ether to precipitate the peptides.

Synthesis of stPERML-R7

After the synthesis of peptide (50 μmol scale) following Method A without deprotecting N-terminus Fmoc group, ring closing metathesis was performed using 20 mol% of second-generation Grubbs catalyst in DMF (2 mL) under N_2 bubbling condition. Final deprotection was performed 2 mL of 20% piperidine DMF solution (v/v) for 20 min. The resin was suspended in cleavage cocktail [2 mL TFA, 50 μL water, 50 μL triisopropylsilane; final concentration: 94% TFA, 2.5% water, 2.5% 1,2-ethanedithiol, 1% triisopropylsilane] for 2 hr at rt. The TFA solution was evaporated to a small volume under a stream of N_2 and dripped into cold ether to precipitate the peptides.

Synthesis of LCL-PERML-R7

After the peptide synthesis of **PERML-R7** moiety (25 μmol scale) following method A, Fmoc-NH-PEG2-CO_2H (4 eq), HBTU (4 eq), DIPEA (4 eq) and HOBt (4 eq) in DMF (2 mL) were added, and the reaction mixture was shaken for 1 hr. After deprotection of the Fmoc groups and repeating the above reaction, the N-Boc protected LCL161 (2 eq) was coupled with the peptide using HBTU (2 eq), DIPEA (4 eq), and HOBt (2 eq) in DMF (2 mL). The resin was suspended in cleavage cocktail [2 mL TFA, 50 μL water, 50 μL 1,2-ethanedithiol, 20 μL triisopropylsilane; final concentration: 94% TFA, 2.5% water, 2.5% 1,2-ethanedithiol, 1% triisopropylsilane] for 2 hr at rt. The TFA solution was evaporated to a small volume under a stream of N_2 and dripped into cold ether to precipitate the peptides.

Synthesis of PERML (R)

Employing the procedure described above for **PERML** and starting from **PERML**.

Synthesis of LCL-stPERML-R7

Employing the procedure described above for **LCL-PERML-R7** and starting from **stPERML-R7** afforded **LCL-stPERML-R7**.

Synthesis of FAM-PERML

After the peptide synthesis of **PERML** moiety (25 μmol scale) following Method A, Fmoc-βAla-OH (4 eq), HBTU (4 eq), DIPEA (8 eq), HOBt (4 eq) in DMF (2 mL) were added, and the reaction mixture was shaken for 1 hr. After deprotection of the Fmoc groups, the 5,6-carboxyfluorescein (4 eq) was coupled with the peptide using HBTU (4 eq), DIPEA (8 eq), and HOBt (4 eq) in DMF (2 mL). The resin was suspended in cleavage cocktail [2 mL TFA, 50 μL water, 50 μL 1,2-ethanedithiol, 20 μL triisopropylsilane; final concentration: 94% TFA, 2.5% water, 2.5% 1,2-ethanedithiol, 1% triisopropylsilane] for 2 hr at rt. The TFA solution was evaporated to a small volume under a stream of N_2 and dripped into cold ether to precipitate the peptides.

Characterization of Peptides

Peptide purity was checked by analytical HPLC using Discovery® BIO Wide Pore C18 column (25 cm x 4.6 mm).

solvent A; 0.1% TFA/water

solvent B; 0.1% TFA/MeCN

flow rate; 1.0 mL/min

gradient; 10-90% gradient of solvent B over 30 min

PERML

HPLC: Conditions = solvent A 0.1% TFA/water, solvent B 0.1% TFA/CH_3CN

Gradient = 10-90% gradient of solvent B over 40 min

Purity: 100% (t_R = 8.76 min)

HRMS (ESI^+) calcd for $C_{44}H_{86}N_{16}O_9S$ $[M+2H]^{2+}$: 507.3237; found: 507.3238

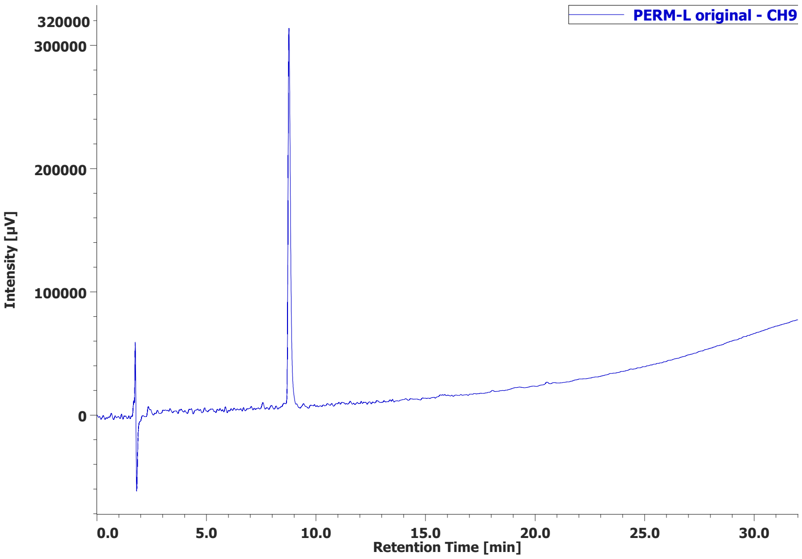

HPLC spectrum of purified **PERML**

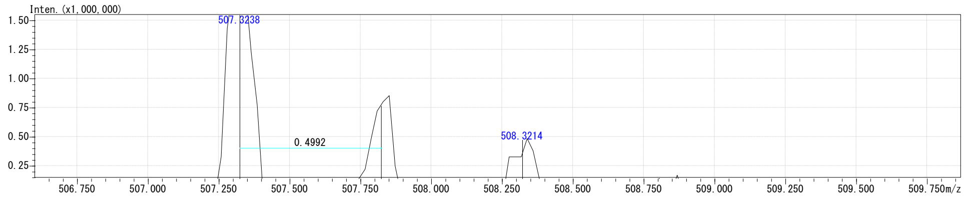

PERML-R7:

HPLC: Conditions = solvent A 0.1% TFA/water, solvent B 0.1% TFA/CH_3CN

Gradient = 10-90% gradient of solvent B over 40 min

Purity: 96.8% (t_R = 8.76 min)

HRMS (ESI^+) calcd for $C_{92}H_{177}N_{45}O_{19}S$ $[M+2H]^{4+}$: 563.1070; found: 563.1077

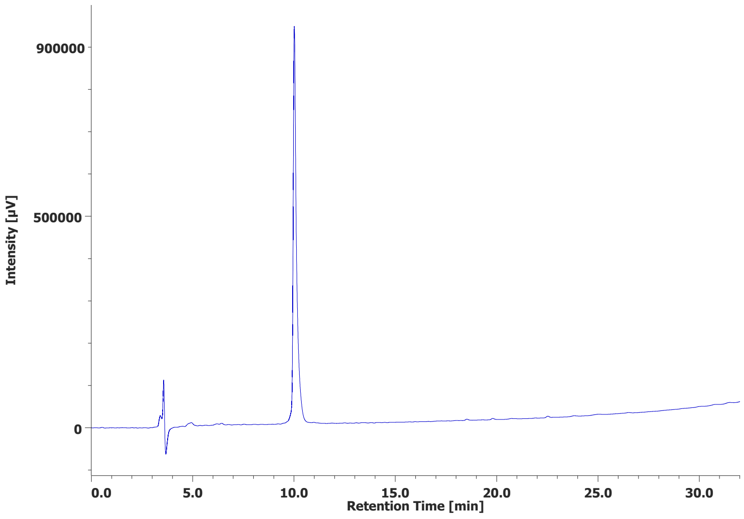

HPLC spectrum of purified **PERML-R7**

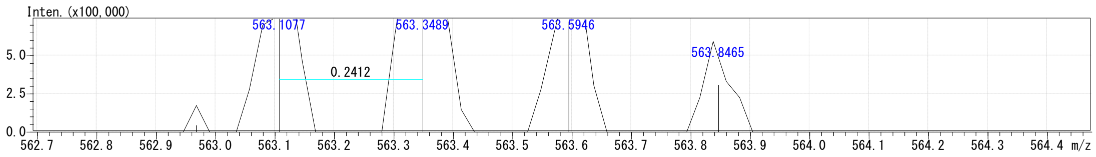

stPERML

HPLC: Conditions = solvent A 0.1% TFA/water, solvent B 0.1% TFA/CH_3CN

Gradient = 20-60% gradient of solvent B over 40 min

Purity: 99.3% (t_R = 14.97 min)

HRMS (ESI^+) calcd for $C_{68}H_{102}N_{17}O_{16}S$ $[M+2H]^{2+}$:566.9001; found: 566.8438

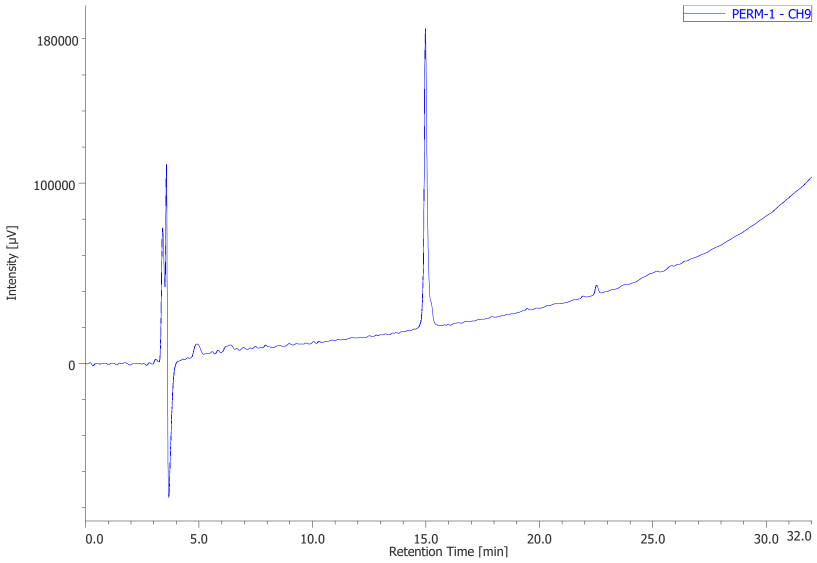

HPLC spectrum of purified **stPERML**

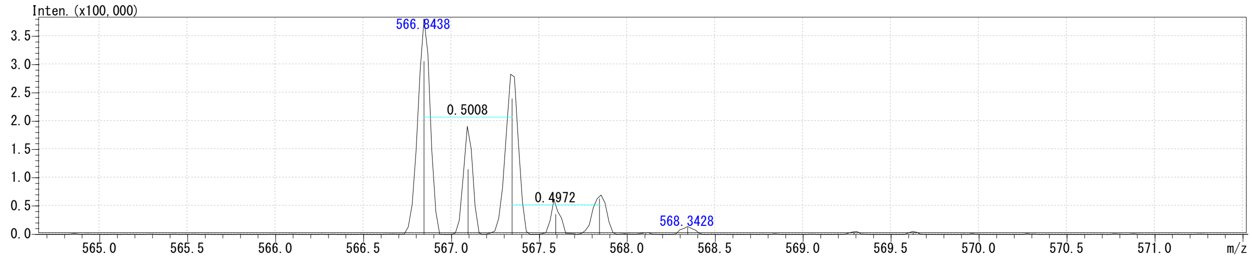

stPERML-R7:

HPLC: Conditions = solvent A 0.1% TFA/water, solvent B 0.1% TFA/CH_3CN

Gradient = 10-90% gradient of solvent B over 40 min

Purity: 91.6% (t_R = 14.84 min)

HRMS (ESI^+) calcd for $C_{103}H_{199}N_{46}O_{20}$ $[M+5H]^{5+}$: 488.5209; found: 488.5193

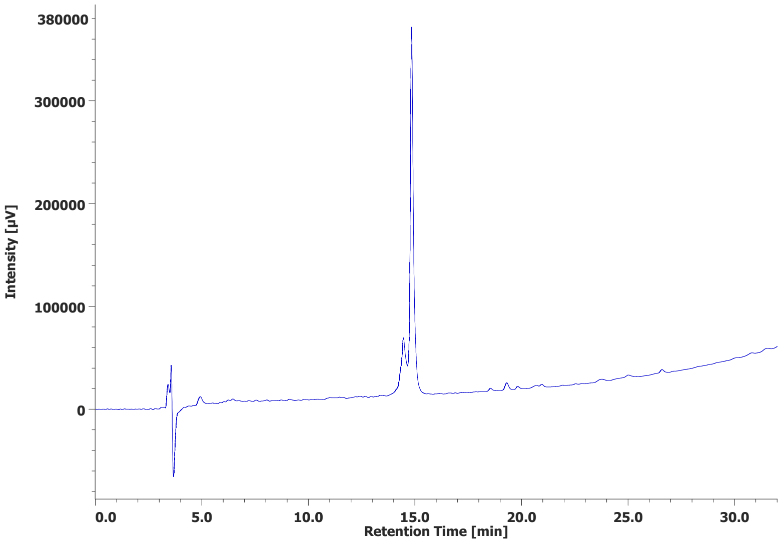

HPLC spectrum of purified **stPERML-R7**

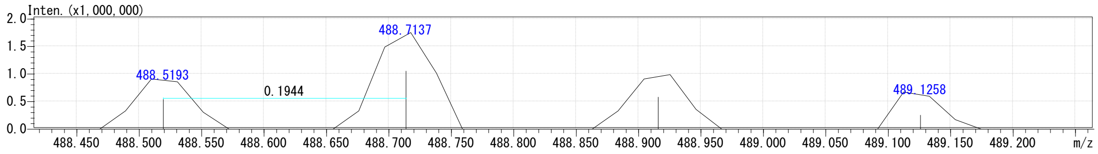

LCL-PERML-R7:

HPLC: Conditions = solvent A 0.1% TFA/water, solvent B 0.1% TFA/CH_3CN

Gradient = 10-90% gradient of solvent B over 40 min

Purity: 87.4% (t_R = 10.19 min)

HRMS (ESI^+) calcd for $C_{132}H_{233}N_{51}O_{30}S_2$ $[M+7H]^{7+}$: 440.5461; found: 440.5465

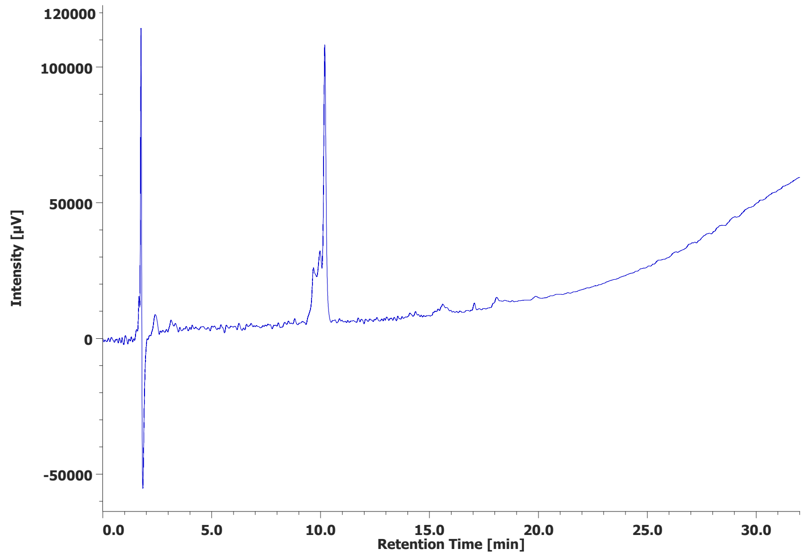

HPLC spectrum of purified **LCL-PERML-R7**

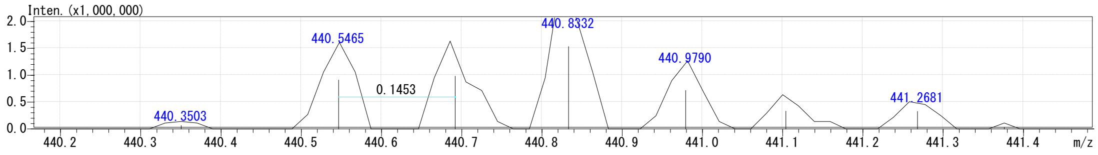

LCL-stPERML-R7:

HPLC: Conditions = solvent A 0.1% TFA/water, solvent B 0.1% TFA/CH_3CN

Gradient = 20-40% gradient of solvent B over 40 min

Purity: 97.8% (t_R = 12.12 min)

HRMS (ESI^+) calcd for $C_{143}H_{250}N_{59}O_{31}S$ $[M+7H]^{7+}$: 461.5688; found: 461.5693

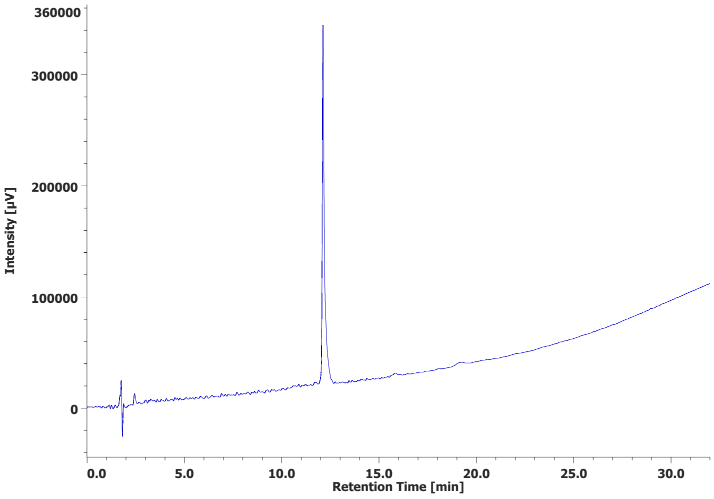

HPLC spectrum of purified **LCL-stPERML-R7**

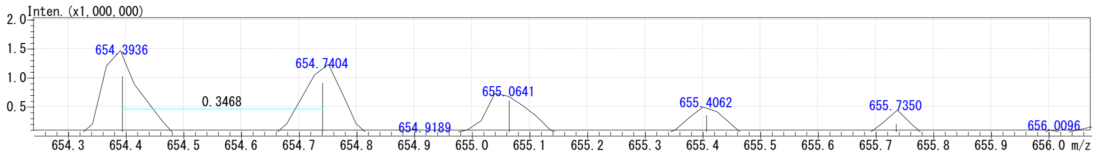

FAM-PERML

Sequence: FAM-(β-Ala)-Arg-Ile-Leu-Arg-Cys-Leu-Lue-Gln-NH_2

HPLC: Conditions = solvent A 0.1% TFA/water, solvent B 0.1% TFA/CH_3CN

Gradient = 10-90% gradient of solvent B over 40 min

Purity: 97.8% (t_R = 21.02 min)

HRMS (ESI^+) calcd for $C_{68}H_{102}N_{17}O_{16}S$ $[M+3H]^{3+}$: 481.5798; found: 481.5797

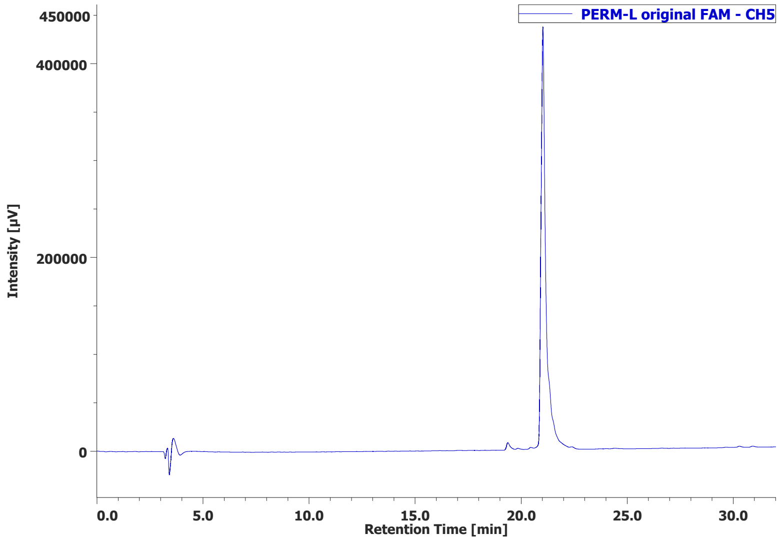

HPLC spectrum of purified **FAM-PERML**

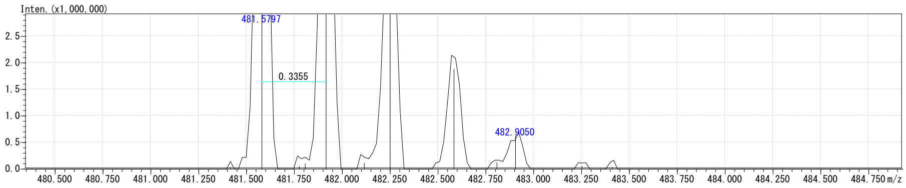

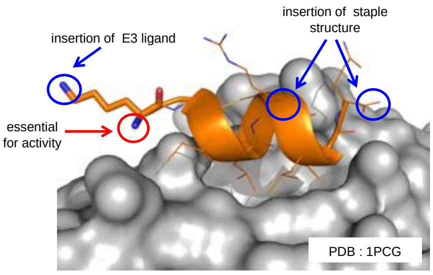

Figure S1. The co-crystal X-ray structure of **PERM** peptide and the ERα.[S1]

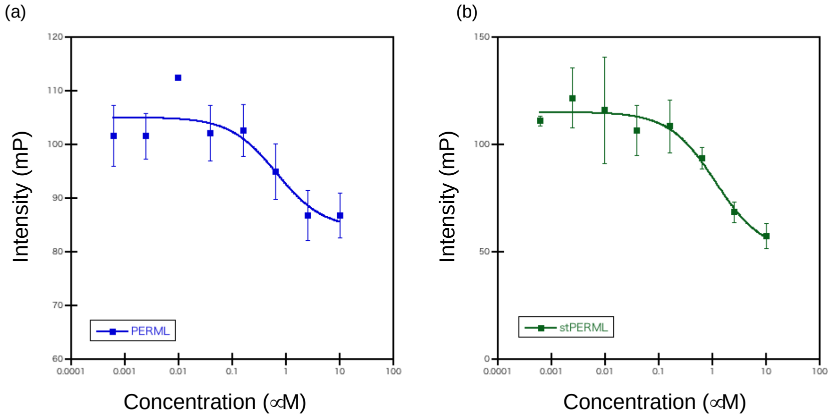

Figure S2. Fluorescence polarization assays of the binding affinity between (a) **PERML** and (b) **stPERML** (n = 3). **PERML** and **stPERML** showed similar affinity toward ERα with IC_{50} values of 0.7 ± 0.7 and 1.1 ± 0.4 µM, respectively.

S1. Leduc, A.M., Trent, J.O., Wittliff, J.L., Bramlett, K.S., Briggs, S.L., Chirgadze, N.Y., Wang, Y., Burris, T.P., Spatola, A.F. *Proc. Natl. Acad. Sci. U.S.A.* **2003**, *100*, 11273-11278.
